# Does individual variation in metabolic phenotype predict fish behaviour and performance?

**DOI:** 10.1111/jfb.12699

**Published:** 2015-11-17

**Authors:** N. B. Metcalfe, T. E. Van Leeuwen, S. S. Killen

**Affiliations:** ^1^ Institute of Biodiversity, Animal Health & Comparative Medicine, Graham Kerr BuildingUniversity of GlasgowGlasgow G12 8QQU.K.; ^2^ Scottish Centre for Ecology and the Natural Environment (SCENE)University of Glasgow, RowardennanLoch LomondGlasgow G63 0AWU.K.

**Keywords:** aerobic scope, dominance, fitness, growth, metabolism, specific dynamic action

## Abstract

There is increasing interest in documenting and explaining the existence of marked intraspecific variation in metabolic rate in animals, with fishes providing some of the best‐studied examples. After accounting for variation due to other factors, there can typically be a two to three‐fold variation among individual fishes for both standard and maximum metabolic rate (SMR and MMR). This variation is reasonably consistent over time (provided that conditions remain stable), and its underlying causes may be influenced by both genes and developmental conditions. In this paper, current knowledge of the extent and causes of individual variation in SMR, MMR and aerobic scope (AS), collectively its metabolic phenotype, is reviewed and potential links among metabolism, behaviour and performance are described. Intraspecific variation in metabolism has been found to be related to other traits: fishes with a relatively high SMR tend to be more dominant and grow faster in high food environments, but may lose their advantage and are more prone to risk‐taking when conditions deteriorate. In contrast to the wide body of research examining links between SMR and behavioural traits, very little work has been directed towards understanding the ecological consequences of individual variation in MMR and AS. Although AS can differ among populations of the same species in response to performance demands, virtually nothing is known about the effects of AS on individual behaviours such as those associated with foraging or predator avoidance. Further, while factors such as food availability, temperature, hypoxia and the fish's social environment are known to alter resting and MMRs in fishes, there is a paucity of studies examining how these effects vary among individuals, and how this variation relates to behaviour. Given the observed links between metabolism and measures of performance, understanding the metabolic responses of individuals to changing environments will be a key area for future research because the environment will have a strong influence on which animals survive predation, become dominant and ultimately have the highest reproductive success. Although current evidence suggests that variation in SMR may be maintained within populations via context‐dependent fitness benefits, it is suggested that a more integrative approach is now required to fully understand how the environment can modulate individual performance via effects on metabolic phenotypes encompassing SMR, MMR and AS.

## Introduction

Fishes have provided some of the best examples of intraspecific variation in morphological features that reveal adaptations to local environments. These include differences in gill‐raker spacing and mouth shape as adaptations to feeding on benthic *v*. planktonic resources (Schluter, 1993), changes in body shape that reduce the risk of predator attack (Brönmark & Miner, 1992) or increase the ability to escape (Walker, 1997) and variation in shape related to habitat differences in water speed (Peres‐Neto & Magnan, 2004). Recently, however, it has become clear that more cryptic physiological measures may be equally important in explaining patterns of adaptation and life‐history diversity within species. The increasing interest in the role of physiology in shaping evolution (Irschick & Garland, 2001; Irschick *et al.*, 2008) and in mediating life‐history trade‐offs (Zera & Harshman, 2001; Monaghan *et al.*, 2009) has focussed attention on individual variation in physiological traits. Such traits have historically been more poorly documented than morphological features. Recent technological and analytical advances [*e*.*g*. the development of oxygen sensing software and technology (optodes) for respirometry of aquatic organisms (Nelson, 2016)], however, have made it increasingly possible to obtain data from sufficient numbers of individuals to quantify the level of intraspecific variation in a range of physiological traits. This has led to a relatively new approach to ecophysiology which is focused on understanding the causes and ecological consequences of individual variability in physiological traits, rather than treating it as noise or error between measurements.

One of the main physiological traits of interest in this study of intraspecific variation has been metabolic rate, partly because it can be measured using non‐invasive means and partly because it is assumed to have broad ecological relevance (Burton *et al.*, 2011b). Standard metabolic rate (SMR), which is equivalent to basal metabolic rate (BMR) in endotherms, is the minimal maintenance metabolic rate of an ectotherm in a post‐absorptive and inactive state (Chabot *et al.*, 2016a). SMR (usually measured in terms of oxygen consumption) is an integrated measure of the physiological energy expenditures involved in the anabolism and catabolism of tissues and organism homeostasis. After controlling for temperature and body size, SMR varies among fish species in relation to their lifestyle (*e*.*g*. benthic *v*. pelagic) (Killen *et al.*, 2010). After controlling for these sources of variation, however, MR often differs by a factor of up to two or three among individual fish of the same age, sex and species held in similar conditions (Millidine *et al.*, 2009; Norin & Malte, 2011; Killen *et al.*, 2012a). The related term routine metabolic rate (RMR) refers to the average rate of metabolism when the animal is undergoing normal behaviours or some other specified type of activity (although these should be carefully defined). The term resting metabolic rate, although somewhat vague and not ideal when applied to fishes, is sometimes used when interchangeably discussing both SMR and RMR. Aerobic scope (AS) is defined as the difference between an animal's SMR and its maximum possible aerobic metabolic rate (MMR) under the same environmental conditions, so that AS defines the capacity of the animal to increase its rate of aerobic metabolism (Norin & Clark, 2016). Together, these metabolic traits make up what will be referred to as the metabolic phenotype of the individual. The focus will mostly be on SMR, MMR and AS as their meaning is more precisely defined. While there have been fewer studies of MMR and AS than there have been of SMR, it would appear that MMR and AS show similar levels of intraspecific variation as SMR (Norin & Malte, 2011, 2012; Killen *et al.*, 2012a; Auer *et al.*, 2015a).

The extent of this intraspecific variation in metabolic rate is in line with that found in other taxa (Steyermark *et al.*, 2005; Johnston *et al.*, 2007). Given the profound consequences that this has for energy budgets, its persistence demands explanation and has been the subject of a number of recent reviews that attempt to link intraspecific variation in minimal or maximal metabolism to other biological traits or ecological factors (Gomes *et al.*, 2004; Biro & Stamps, 2010; McKechnie & Swanson, 2010; Burton *et al.*, 2011b; Konarzewski & Ksiazek, 2013). Here, the aim is to provide a synopsis on the current understanding of intraspecific variation in these various components of the metabolic phenotype and their links to other traits in fishes, by specifically focusing on (1) the extent and causes of individual variation and repeatability of metabolic traits, (2) how the metabolic phenotype varies with environmental conditions (food availability, temperature, hypoxia, salinity and shelter) and (3) the relationships between the metabolic phenotype and other whole‐organism traits (such as dominance, aggression, risk‐taking, boldness and swimming performance). In conclusion, several key areas are outlined where it is felt that information is currently lacking. The goal is to understand how this individual variation in metabolism arises and persists by considering its consequences under different ecological conditions.

## Individual variation in metabolism

### The consistency of individual variation in metabolic rate

If measurements of metabolic rate are taken at only a single point in time (as is the case in the great majority of studies), there is the risk that the observed variation between individuals is simply due to measurement error or random temporal fluctuations in metabolism. There have now been enough studies that have taken repeated measurements from the same individuals, however, to allow a proper evaluation of the consistency of these traits over time. The initial studies showed that individual differences in SMR tended to be repeatable: measures of relative SMR (*i*.*e*. after correcting for differences in body mass) in the same fish but at different times were found to be significantly correlated, with correlation coefficients ranging from 0·40 (O'Connor *et al.*, 2000) to 0·68 in Atlantic salmon *Salmo salar* L. 1758 (McCarthy, 2000). Significant repeatabilities were also found in individual Arctic charr *Salvelinus alpinus* (L. 1758) measured at intervals of up to 6 months (Cutts *et al.*, 2001).

This body of work, together with a meta‐analysis of metabolic rates across the vertebrates (Nespolo & Franco, 2007), led to the general consensus that minimal rates of metabolism were stable traits of individuals. This has been supported by more recent studies of other fish species [*e*.*g*. correlation coefficients of 0·68–0·73 in spined loach *Cobitis taenia* L. 1758 (Maciak & Konarzewski, 2010), 0·71 in *S*. *alpinus* (Voutilainen *et al.*, 2011) and 0·50 in European eel *Anguilla anguilla* (L. 1758) (Boldsen *et al.*, 2013)]. This view has recently been challenged by new evidence suggesting that the repeatability decreases over time (Norin & Malte, 2011; White *et al.*, 2013). The highest correlations between repeated assessments of an individual's SMR occur when the time interval between the two measurements is short [the most extreme case being *r* = 0·88 for bleak *Alburnus alburnus* (L. 1758) when the two respirometry trials were separated by only an hour (Voutilainen *et al.*, 2011)]. As the interval between trials increases, the measurements of relative SMR become less similar. The most detailed analysis of this effect comes from the study of brown trout *Salmo trutta* L. 1758 by Norin & Malte (2011), who demonstrated a decline in correlation coefficients from 0·57 to 0·09 as the time interval between measurements increased from 35 to 105 days. The rate at which the repeatability decreases is not constant, relative SMR was found to remain very consistent (*r* = 0·68) over 17 weeks in juvenile *S*. *salar* (despite a 20‐fold average increase in their body size) (McCarthy, 2000). In contrast, slightly older individuals of the same species were found to exhibit no repeatability in relative SMR when measurements were separated by 6 months (*r* = 0·02) or 14 months (*r* = −0·17), although there was a significant correlation at an interval of 8 months (*r* = 0·51) (Seppänen *et al.*, 2010). It is worth noting, however, that the only studies examining temporal changes in repeatability in fishes have been conducted on populations held under homogeneous laboratory conditions. It is therefore possible that in this situation behavioural feedbacks associated with food acquisition, habitat choice or dominance hierarchies, all of which may affect the metabolic rate of individuals, are largely prevented from exerting effects that could otherwise preserve repeatability over longer temporal scales. More work is therefore needed to examine the repeatability of metabolic traits in semi‐natural or natural conditions.

Changes in environmental conditions or the physiological state of the fish may also act to erode repeatability in metabolic rate: no correlation was found between the relative SMR of juvenile *S*. *salar* when measured on *ad libitum* rations and when measured after having been deprived of food for 3 weeks, although the correlation was restored (*r* = 0·40 between initial and final measurement) once the fish had been back on *ad libitum* food for 4 weeks (O'Connor *et al.*, 2000). Similarly, the correlations between repeated measurements of relative SMR in barramundi *Lates calcarifer* (Bloch 1790) were weaker when comparing tests carried out in contrasting environments (*e*.*g*. differing temperatures, salinities or levels of hypoxia) compared with the same environment (Norin *et al.*, 2015).

There have been far fewer studies of the within‐individual repeatability of MMR or AS, but to date these show the same trends as for SMR. Thus, studies of *S*. *trutta*, *A*. *alburnus* and *S*. *alpinus* suggest that there is a significant repeatability in MMR and AS that tends to decline with increasing time intervals between measurements, at least in the simplified environment of the laboratory (Norin & Malte, 2011; Voutilainen *et al.*, 2011), and also declines with a change in environmental conditions (Norin *et al.*, 2015).

### The underlying causes of individual variation in metabolism

There has been speculation over the physiological differences that generate intraspecific variation in metabolic rate, but even when considering other taxa the picture is not clear, whether the metabolism is minimal (Burton *et al.*, 2011b; Konarzewski & Ksiazek, 2013) or maximal (Gebczynski & Konarzewski, 2009). A number of studies reviewed by Konarzewski & Ksiazek (2013) have shown that both interspecific and intraspecific variation in body size‐corrected BMR is positively associated with variation in the relative size of metabolically expensive organs such as the heart, liver and brain. Relationships between organ size and metabolic rate appear to be less evident in fishes, however, possibly because the key organs make up a smaller proportion of the total body size in fishes compared with endotherms. While relative liver size was found to be a predictor of relative SMR in *A*. *anguilla* (Boldsen *et al.*, 2013), neither SMR nor MMR were related to the sizes of any of the key organs in *S*. *trutta* (Norin & Malte, 2012). Instead, intraspecific variation in both SMR and (to a lesser extent) MMR in the latter species were positively correlated with the activity of two key aerobic mitochondrial enzymes (cytochrome C oxidase and citrate synthase) (Norin & Malte, 2012). Negative relationships between SMR and erythrocyte size have also been found in individual *C*. *taenia* differing in ploidy levels (Maciak *et al.*, 2011), but while variation in cell size may explain some of the interspecific variation in metabolic rate, the uniformity of cell size in most species makes it unlikely that this is a major contributor to variation in metabolic rate within species.

Less is known about the factors influencing MMR. Cross‐species analyses suggest that MMR is constrained by the mechanics of both oxygen and carbon dioxide transport (Hillman *et al.*, 2013). Several studies have documented correlations among SMR, MMR and AS across individuals. The existence of these associations suggests a functional link among these traits, but the direction of the correlations vary among species. Positive correlations have been observed between SMR and MMR for grass carp *Ctenopharyngodon idella* (Valenciennes 1844) (Zhang *et al.*, 2014) and *S*. *trutta* (Norin & Malte, 2012), and between resting metabolic rate and MMR following feeding in southern catfish *Silurus meridionalis* Chen 1977 (Fu *et al.*, 2005). In contrast, SMR and measures of AS are negatively correlated in the common minnow *Phoxinus phoxinus* (L. 1758) (Killen, 2014) and juvenile *S*. *salar* (Cutts *et al.*, 2002). The nature of the relationships among these traits may depend on the species in question and the exact metabolic indices measured. Further, it is possible that the pattern of energy budgeting and allocation within species can affect correlations among traits within individuals.

A separate question is whether these differences are primarily under genetic or environmental control. While there are very few studies of the genetics of metabolic rate in fishes, mass‐independent BMR has been shown to be heritable in birds (Mathot *et al.*, 2013) and mammals (Zub *et al.*, 2012; Boratynski *et al.*, 2013), as has MMR (Wone *et al.*, 2009), and there is evidence of a paternal effect on SMR in *S*. *salar* (Pakkasmaa *et al.*, 2006). SMR has also been found to differ between individuals from different populations of *S*. *salar* and *S*. *trutta*, even when reared in a common environment (Lahti *et al.*, 2002; Seppänen *et al.*, 2009a, b), which again suggests a genetic component to intraspecific variation in metabolic rate in fishes.

It is clear that metabolism can be influenced by maternal (*i*.*e*. non‐genetic) effects (Régnier *et al.*, 2010): the RMR of *S*. *salar* fry and the relative SMR of *S*. *trutta* embryos were both found to decrease with increasing egg size (Rossignol *et al.*, 2010; Régnier *et al.*, 2012), while Burton *et al.* (2013) found SMR in *S*. *trutta* fry to vary according to the position that the egg occupied in the mother's ovary (with stronger positional effects in more dominant mothers). The reason for these effects is not known, but may relate to variation in the composition of the eggs: female fish deposit hormones into their eggs, and manipulations of egg cortisol levels have been found to influence the SMR of the resulting offspring in *S*. *trutta* (Sloman, 2010) [although a slightly different method of manipulating cortisol levels in *S*. *trutta* eggs found no such effect (Burton *et al.*, 2011a)].

### Individual variation in metabolic responses to feeding and food availability

Specific dynamic action (SDA), the increase in organismal metabolism as a result of food ingestion (Chabot *et al.*, 2016b), can constitute upwards of 60–80% of the maximum rate of oxygen consumption (Alsop & Wood, 1997) or two to three times the baseline metabolic expenditure and in fishes can last anything from 3 to 390 h (Secor, 2009). In some sedentary species, the peak in oxygen uptake following feeding can exceed that observed during peak aerobic exercise (Fu *et al.*, 2005). Although much of the variation in duration and peak SDA can be explained by differences in fish size and species, ambient temperature and composition of a given meal (Secor, 2009), there remains considerable variation that can probably be attributed to inherent differences between individuals. For example, in both *S*. *meridionalis* and *S*. *salar*, it has been shown that individuals with a relatively high SMR for their size had a higher peak and overall magnitude of SDA but had a shorter duration of the SDA response, indicating that the metabolism of these individuals was quicker to return to resting levels after each meal compared with low SMR individuals (Fu *et al.*, 2005; Millidine *et al.*, 2009). Based on these results, it was thought that individuals with a high SMR and consequently a shorter SDA response may have a growth advantage under high food conditions, since a strategy based on quick ingestion and assimilation may allow for greater throughput of food and ultimately growth; high SMR fishes may also be more dominant and so have priority of access to food. It has recently been shown in *S. trutta* that under conditions of *ad libitum* food the amount consumed per day is positively related to AS (Auer *et al.*, 2015c). Under conditions of low food availability, however, this strategy may become maladaptive due to the higher energy demands placed on individuals with a high SMR, which is not offset by any increased food intake. It should be noted that the rate of growth may itself influence the estimate of SMR, depending on the protocol that is used (Rosenfeld *et al.*, 2015), which may help explain inconsistencies found between studies that relate individual differences in metabolic rate to differences in growth rate (Hoogenboom *et al.*, 2013). Laboratory studies (in which food is generally supplied *ad libitum*) have generally found a positive correlation between SMR and growth rate (Cutts *et al.*, 1998; Yamamoto *et al.*, 1998), whereas equivalent analyses carried out on fishes in conditions where food is more likely to be limiting (*e*.*g*. the natural environment) have found either negative or no correlations (Álvarez & Nicieza, 2005; Finstad *et al.*, 2007a; Norin & Malte, 2011; Robertsen *et al.*, 2015). It has been suggested that the fitness advantages of a given SMR may thus depend upon the context (Burton *et al.*, 2011b), a hypothesis that is supported by recent stream‐tank experiments showing that SMR is positively correlated with growth rate when fishes are competing for access to a plentiful food supply but the relationship becomes negative once access to food is taken into account (Reid *et al.*, 2011, 2012).

While the direct energetic consequences of SDA are not included as part of SMR, MMR or AS (and are controlled for by fasting individuals prior to measurement), the indirect effects of the metabolically expensive organs associated with shaping the SDA response may play a pivotal role in explaining individual differences in metabolism (Rosenfeld *et al.*, 2015). What remains inconsistent, however, is whether the size of metabolically expensive organs associated with ingestion, digestion and assimilation (*i*.*e*. stomach, liver, heart and intestine) drives the individual differences in baseline metabolism. For example, as mentioned earlier, no relationship between variation in metabolism (SMR, MMR and AS) and mass of metabolically active organs was found in *S*. *trutta* (Norin & Malte, 2012) but a positive relationship was found in *A*. *anguilla*, with 38% of the variation in SMR being explained by the mass of internal organs, in particular the liver (Boldsen *et al.*, 2013). Whatever the driver of this relationship, it is clear that individual differences exist in many aspects of the SDA response (*e*.*g*. time to response, peak and duration; Millidine *et al.*, 2009), which will probably have implications for individual variability in the metabolic phenotype irrespective of the routinely applied protocol of fasting fishes prior to respirometry.

Although the SDA response is the most recognizable and documented effect of food on metabolism, the role of food availability in influencing individual variation in metabolic rate independent of SDA should not be ignored. There are numerous studies documenting a decrease in SMR when fishes are subjected to a period of food restriction (Beamish, 1964; Du Preez, 1987; Wieser *et al.*, 1992; Auer *et al.*, 2015b) and an increase in SMR when food is supplied above baseline levels (O'Connor *et al.*, 2000; Van Leeuwen *et al.*, 2011, 2012; Auer *et al.*, 2015b). While there have been few studies investigating the effect of food level on MMR and AS, those that exist suggest that food level has contrasting effects on MMR and AS. Van Leeuwen *et al.* (2011) found no relationship between food level and MMR in juvenile steelhead *Oncorhynchus mykiss* (Walbaum 1792) or coho salmon *Oncorhynchus kisutch* (Walbaum 1792) but did find a negative relationship between food availability and AS. In addition, *P*. *phoxinus* that experienced a period of food deprivation and subsequent compensatory growth were observed to have an elevated SMR and reduced AS compared with fish that had been feeding *ad libitum* throughout the entire period, although there was no change in MMR (Killen, 2014).These results suggest that an individual's MMR may be relatively uninfluenced by the fish's nutritional state and that AS is primarily driven by the degree of plasticity within an individual's SMR.

Individual fish that show the greatest flexibility in SMR in response to changing food availability have the fastest growth (Auer *et al.*, 2015b). What remains unclear is what processes are responsible for the changes in SMR with varying food conditions. SMR is the culmination of a large number of background processes that consume oxygen (Darveau *et al.*, 2002), and so the variation in SMR due to nutrition could be due to changes in cell maintenance, cell growth and organ mass, but there may also be changes in an individual's investment in energetically costly tissues related to its ability to obtain food (*e*.*g*. its lifestyle, level of locomotor performance and competitive ability). This warrants further investigation, but it is clear that an individual's nutritional condition must be taken into account when undertaking measurements of SMR and AS.

### Individual variation in metabolic responses to abiotic factors

Relationships between metabolic rate and temperature have formed the basis for some of the most fundamental and debated general theories in ecology, ranging from the metabolic theory of ecology (Gillooly *et al.*, 2001) to the more recently proposed metabolic‐level boundaries hypothesis (Glazier, 2010), with incorporation of an organism's lifestyle (Killen *et al.*, 2010), and the oxygen and capacity limited thermal tolerance (OCLTT) hypothesis (Pörtner & Knust, 2007; Norin & Clark, 2016). While the effects of temperature on the metabolism of fishes have been well documented (Fry, 1971), the great majority of studies have ignored individual variation in that response, the available evidence suggests that not all members of a population have the same reaction norm. An individual's relative SMR, MMR or AS at one temperature is therefore not a precise predictor of its metabolism if the temperature changes (Norin *et al.*, 2015).

As with temperature, the metabolic response to hypoxia is generally context dependent (Claireaux & Chabot, 2016). MMR and AS are likely to be more affected by hypoxia than is SMR, given that depleted oxygen concentrations are more likely to affect the maximum performance of the cardiovascular system than baseline metabolism. Dupont‐Prinet *et al.* (2013) found that acute hypoxia did not affect SMR in juvenile Greenland halibut *Reinhardtius hippoglossoides* (Walbaum 1792) but did significantly reduce MMR and AS, consistent with other studies across fishes (Petersen & Gamperl, 2010; Norin *et al.*, 2015). Given the fundamental role of metabolism in mediating the response to hypoxia and the variability in individual metabolism, it is likely that individuals with a higher or lower metabolic rate will respond differently (both physiologically and behaviourally) to hypoxia exposure. Norin *et al.* (2015) found that MMR and AS decreased under hypoxia, but that low MMR and AS individuals were much less affected than their high MMR and AS counterparts. These results suggest that individuals with a higher aerobic capacity (MMR and AS) are more likely to be constrained by decreases in oxygen compared with low metabolic rate individuals. Therefore, hypoxia may play a significant role in shaping individual personality, although the ecological consequences of these results in the wild are relatively unknown. Results from a laboratory study conducted by Killen *et al.* (2012b) found a positive correlation between SMR and activity, risk‐taking and aquatic surface respiration (ASR) in European sea bass *Dicentrarchus labrax* (L. 1758), but only under conditions of severe hypoxia. The results suggested that as oxygen levels in water decline, high SMR individuals are the first to be forced to expose themselves to greater predation risk to counteract the decrease in oxygen concentration (Killen *et al.*, 2012b). This study supports the work of Domenici *et al.* (2007) who suggested that a trade‐off may exist between fishes undergoing ASR to avoid the detrimental effects of hypoxia and vulnerability to aerial predation.

The movement of osmotic solutes across the osmotic gradient is generally regarded as being energetically expensive, and in extreme cases may constitute upwards of 20–50% of the total energy budget in fishes (Boeuf & Payan, 2001). Fishes in freshwater environments tend to have a lower energetic cost associated with osmoregulation (as revealed by a lower SMR) compared with those in full‐strength salt water (Morgan & Iwama, 1991; Kitano *et al.*, 2010), although other studies comparing three‐spined sticklebacks *Gasterosteus aculeatus* L. 1758 from populations experiencing differing osmoregulatory demands have found differences in MMR and AS but not in SMR (Dalziel *et al.*, 2012). Given the degree of interspecific variability in salinity tolerance, it is likely that some intraspecific variation may exist, although studies have been scarce. Norin *et al.* (2015) found that individual *L*. *calcarifer* with a relatively low SMR, MMR and AS showed an increase in their SMR, MMR and AS when salinities were decreased from 35 to 10, whereas fish with relatively high metabolic rates showed a reduction in their SMR and no real change in MMR and AS when exposed to the same decrease in salinity, indicating that changes in salinity will not have the same consequences for metabolic performance in all members of a population.

It is well established that shelter plays a fundamental role in the survival of animals. Not only does it provide a safe refuge from predators, but it also provides a refuge from the environment (Millidine *et al.*, 2006). While the benefits of shelter for fish growth performance is well established (Finstad *et al.*, 2007b), recent evidence suggests that it may also influence the physiology of fishes, even in the absence of predators. The presence of shelter may cause a decrease in the metabolic rate of *S*. *salar* (Finstad *et al.*, 2004; Millidine *et al.*, 2006) and burbot *Lota lota* (L. 1758) (Fischer, 2000), although no such relationship was found in the false clownfish *Amphiprion ocellaris* Cuvier 1830 (Kegler *et al.*, 2013) or the stone loach *Barbatula barbatula* (L. 1758) (Fischer, 2000), indicating that the relationship between shelter and metabolism may be species dependent. It is noteworthy that although the studies demonstrating a reduction in metabolic rate used very different types of shelter [ranging from clear semi‐circular perspex (Millidine *et al.*, 2006) through cobbles (Fischer, 2000) to ice cover (Finstad *et al.*, 2004)], the reduction in metabolism was very similar, being *c*. 30% in the presence of shelter. While the reasons for the observed relationship between shelter and metabolism are unknown, it is thought that the presence of shelter may reduce stress levels or decrease vigilance (a heightened state of body awareness in response to predation), which may in turn elevate opercular ventilation and ultimately metabolism (Millidine *et al.*, 2006).

Given that metabolism has been found to vary considerably among individuals and influence key behavioural traits that will probably determine shelter use such as boldness, dominance and aggression (Metcalfe *et al.*, 1995; Cutts *et al.*, 1998; Killen *et al.*, 2011), it is likely that the metabolic response of individuals to shelter will vary accordingly to their own baseline metabolic rate. One possibility is that individuals with a low SMR will show a larger increase in metabolism in the absence of shelter compared with those with a higher SMR, given that the latter fish tend to be more aggressive and bold.

## Links between the metabolic phenotype and behaviour

### Aggression and dominance

To better understand the ecological relevance of intraspecific variation in metabolic traits, a large research effort has been devoted towards examining the associations between metabolic traits and the behaviour of individual animals (Table I). Of the potential behavioural correlates of metabolic traits in fishes, aggression and dominance have received the most attention. A general observation is that individuals with higher SMR or resting metabolic rates are more aggressive and more likely to become dominant over conspecifics (Metcalfe *et al.*, 1995; Cutts *et al.*, 1998, 1999; Yamamoto *et al.*, 1998; McCarthy, 2001; Sloat & Reeves, 2014), although exceptions have been noted (Killen *et al.*, 2014). Positive correlations between aggression and SMR among populations of the same species have also been observed (Lahti *et al.*, 2002), possibly driven by local variation in factors such as food availability. In contrast, Seppänen *et al.* (2009b) observed variation in SMR among populations of juvenile *S*. *salar* that were not associated with differences in levels of aggression.

**Table I:** Summary of documented relationships between metabolic traits (SMR, standard metabolic rate; RMR, routine metabolic rate; MMR, maximal metabolic rate; AS, aerobic scope) and behaviours or performance in several species of fishes. Positive (+), negative (−) and non‐existent (none) relationships are shown for each pair of traits. Also shown is the life stage at which the data were collected

Species	Life stage	Metabolic trait	Behaviour orperformance	Relationship	Notes	Reference
*Phoxinus phoxinus*	Juvenile	SMR	Activity	None		Killen (2014)
*Phoxinus phoxinus*	Juvenile	AS	Activity	None		Killen (2014)
*Salmo salar*	Juvenile*	RMR	Activity, dispersal	+		Robertsen *et al.* (2015)
*Salmo salar*	Juvenile	SMR	Aggression	+		Cutts *et al.* (1998)
*Salmo trutta*	Juvenile	SMR	Aggression	+	Interpopulation	Lahti *et al.* (2002)
*Salmo salar*	Juvenile	SMR	Aggression	None	Interpopulation	Seppänen *et al.* (2009a, b)
*Oreochromis mossambicus*	Adult	RMR	Aggression	+	Examining costs of aggressive behaviours	Ros *et al.* (2006)
*Neolamprologus pulcher*	Adult	RMR	Aggression, submission	+	Examining costs of aggressive behaviours	Grantner & Taborsky (1998)
*Micropterus salmoides*	Juvenile	SMR	Angling vulnerability	+		Redpath *et al.* (2010)
*Micropterus salmoides*	Juvenile	MMR	Angling vulnerability	+		Redpath *et al.* (2010)
*Micropterus salmoides*	Juvenile	AS	Angling vulnerability	+		Redpath *et al.* (2010)
*Salmo salar*	Juvenile	SMR	Cover use	−		Finstad *et al.* (2007a)
*Oncorhynchus mykiss*	Juvenile	SMR	Dominance	+		McCarthy (2001)
*Oncorhynchus masou*	Juvenile	SMR	Dominance	+		Yamamoto *et al.* (1998)
*Salmo salar*	Juvenile	SMR	Dominance	+	Modulated by prior residence	Cutts *et al.* (1999)
*Pomacentrus amboinensis*	Juvenile	SMR	Dominance	None		Killen *et al.* (2014)
*Pomacentrus amboinensis*	Juvenile	AS	Dominance	+		Killen *et al.* (2014)
*Oncorhynchus mykiss*	Juvenile	SMR	Dominance	+		Sloat & Reeves (2014)
*Salmo salar*	Juvenile	SMR	Dominance	+		Metcalfe *et al.* (1995)
*Salmo salar*	Juvenile	SMR	Dominance	+		Reid *et al.* (2011)
*Salmo salar*	Juvenile	RMR	Dominance	+		Reid *et al.* (2012)
*Oncorhynchus mykiss*	Juvenile	SMR	Early maturation	+/None	Relationship in females but not in males	Sloat & Reeves (2014)
*Salmo salar*	Juvenile	SMR	Early smoltification	+		McCarthy (2000)
*Salmo trutta*	Juvenile	SMR	Emergence time	−		Régnier *et al.* (2012)
*Salmo salar*	Juvenile	RMR	Emergence time	None		Vaz‐Serrano *et al.* (2011)
*Oncorhynchus mykiss*	Juvenile	RMR	Growth	−	Oxygen uptake measured on whole tanks of fish	McKenzie *et al.* (2012)
*Salmo salar*	Juvenile*	RMR	Growth	−		Robertsen *et al.* (2015)
*Salmo salar*	Juvenile	SMR	Growth	− or None	Modulated by food availability and conspecific density	Reid *et al.* (2011)
*Salmo salar*	Juvenile	RMR	Growth	+ or None	Modulated by habitat complexity and food predictability	Reid *et al.* (2012)
*Esox lucius*	Juvenile	Ventilation	Latency to attack prey	+		McGhee *et al.* (2013)
*Cyprinus carpio*	Juvenile	RMR	Risk‐taking	+		Huntingford *et al.* (2010)
*Dicentrarchus labrax*	Juvenile	RMR	Risk‐taking	+ or None	Modulated by hypoxia	Killen *et al.* (2012b)
*Dicentrarchus labrax*	Juvenile	RMR	Risk‐taking	+ or None	Modulated by food‐deprivation	Killen *et al.* (2011)
*Phoxinus phoxinus*	Juvenile	SMR	Temperature p	−		Killen (2014)
*Phoxinus phoxinus*	Juvenile	AS	Temperature preference	None		Killen (2014)

* Metabolic rate measured at the eyed egg stage.

The causal direction of the positive association between aggression and metabolic rate is unclear. Intrinsic differences in metabolic rate may motivate some individuals to be more aggressive to obtain food or territory; conversely, in cases where metabolism has been measured during or shortly after physical interactions, the measurement could be influenced by the activity costs of aggressive behaviours (Neat *et al.*, 1998; Killen *et al.*, 2014). Several studies of cichlid species have found positive relationships between the rate of aggression (whether lateral displays, fin beats or actual attacks) and metabolic rate (Grantner & Taborsky, 1998; Ros *et al.*, 2006; Dijkstra *et al.*, 2013). In addition to the short‐term increases in metabolic rate associated with activity costs during aggression, there can be longer‐term energetic costs associated with social stress causing increase in metabolic rate among submissive individuals (Grantner & Taborsky, 1998; Sloman *et al.*, 2000; Killen *et al.*, 2014).

The majority of work examining links between dominance and metabolic traits in fishes has examined juveniles competing for territories and access to food. The upper limit to metabolic rate is not really relevant in this situation as it is unlikely to be a constraint: the problem for the fish is not the rate at which food can be digested but whether or not the fish can get access to food in the first place. The costs of aggressive behaviours or an intrinsically high metabolic rate can reduce growth when food is patchily distributed (Reid *et al.*, 2011, 2012; Hoogenboom *et al.*, 2012), and the optimal strategy may be to possess a high enough SMR to out‐compete conspecifics but low enough to ensure that excess energy is available for growth in a particular environment. Interestingly, the position of this threshold value for SMR has been observed to increase with population density (Reid *et al.*, 2012).

Despite a general recognition that aggressive behaviour in fishes involves intense exercise (Neat *et al.*, 1998; Seebacher *et al.*, 2013), there have been few studies examining associations between MMR or AS and dominance in fishes. Killen *et al.* (2014) observed that for juvenile Ambon damselfish *Pomacentrus amboinensis* Bleeker 1868 competing for coral reef territories, dominance status was positively related to AS but not to resting metabolic rate. The mechanism underlying this link between AS and aggression in the species is unclear because individuals in this species do not routinely approach maximal rates of oxygen consumption during agonistic encounters. In contrast, the increase in oxygen consumption resulting from aggressive behaviours in female adult eastern mosquito fish *Gambusia holbrooki* Girard 1859 can occupy the entire AS of an individual (Seebacher *et al.*, 2013). Repeated bouts of conflict may even elicit a training effect by which individuals alter their metabolic traits. For example, in response to repeated chasing and harassment by males over several months, female guppies *Poecilia reticulata* Peters 1859 increase swimming efficiency and thus increase available AS while moving at a given speed as compared with females that have not been exposed to harassment (Killen *et al.*, 2015).

Despite the volume of studies examining links between dominance and metabolic traits in fishes, there is still much more work to be done in this area. For example, most research to date has examined the role of metabolic rates during dyadic contests. Such contests may be inaccurate representations of social structures in nature, which will more likely consist of a network of interactions among individuals within a common shoal or with neighbouring territories (Sloman & Armstrong, 2002). Future work should examine the role of metabolic traits within dominance hierarchies within social networks (Croft *et al.*, 2004), or the establishment of territories within a habitat matrix with multiple interacting conspecifics.

### Risk‐associated behaviours

The energy demand of individuals should be intimately related to the ways in which they obtain food from their environment, and potentially, the risks they are willing to take to get that food. As a result, metabolic rate could not only be related to foraging behaviours but also to traits that increase the risk of being captured by a predator, including boldness and spontaneous activity level. Individuals that display intrinsically high levels of activity may also develop an increased physiological capacity to facilitate increased rates of movement (Biro & Stamps, 2010). Correlations between MMR or AS and behaviours associated with foraging or risk‐taking could therefore emerge, although as far as is known, no study has examined these potential links directly.

Consistent with observations made on other taxa, work with fishes has shown positive correlations between measures of resting metabolic rate, SMR and boldness or activity level among individuals. For example, common carp *Cyprinus carpio* L. 1758 that display the riskiest behaviour have a higher resting metabolic rate when compared to those that take fewer risks (Huntingford *et al.*, 2010). Krause *et al.* (1998) demonstrated that *G*. *aculeatus* that lost the most body mass during food deprivation (a proxy for metabolic energy expenditure) also showed greater reductions in the time until emergence from cover after startling when tested before and after food deprivation. For *S*. *salar* fry, individuals that had moved furthest from their nest site at the time of capture had a higher metabolic rate as measured during the egg stage (Robertsen *et al.*, 2015). Largemouth bass *Micropterus salmoides* (Lacépède 1802) selected for a high vulnerability to angling, a trait which is also known to be linked to boldness and aggression (Biro & Post, 2008; Sutter *et al.*, 2012; Klefoth *et al.*, 2013), show higher SMR and MMR as compared with individuals selected for low angling vulnerability (Redpath *et al.*, 2010). Despite the general observation that metabolic traits often correlate positively with activity, boldness and exploratory behaviour, there are exceptions. For example, when measured in the field, brook charr *Salvelinus fontinalis* (Mitchell 1814) display no link between resting metabolic rate and spontaneous activity (Farwell & McLaughlin, 2009).

There are several possible sources of discrepancies among studies in terms of the strength of correlations between metabolic traits and risk‐associated behaviours. Firstly, environmental conditions may modulate the strength of correlations by altering intraspecific variation in the traits being examined (Killen *et al.*, 2013). *Dicentrarchus labrax*, for example, show no evidence of links between resting metabolic rate and risk‐taking when tested under benign conditions, but a positive correlation emerges among individuals after a period of food deprivation or exposure to hypoxia (Killen *et al.*, 2011, 2012b). Related to this point, it may be possible for correlations to arise depending on whether traits are measured under all environmental contexts under consideration, or if instead behaviours measured under one condition are being compared to metabolic traits measured under another (Killen *et al.*, 2013). Secondly, differences in morphological defences (*e*.*g*. spines) against predation among species may alter the relative costs of reduced foraging opportunities (Krause *et al.*, 2000), possibly generating species‐specific differences in co‐variation between metabolic traits and risk‐taking behaviour. Thirdly, correlations between metabolism and traits such as boldness may be masked by differential responses to the stress imposed during measurement of metabolic rates by bold and shy phenotypes. For example, shy individuals may show increased oxygen consumption when measured in respirometers due to high sensitivity to handling and confinement stress (Martins *et al.*, 2011).

### Foraging ability

Most studies examining behavioural correlates between metabolic traits and foraging have focussed on the perspective of the prey, evaluating the tendency to take risks in relation to individual metabolic rates. Studies examining links to foraging success are rare, but the attack latency of predatory pike *Esox lucius* L. 1758 is positively correlated with resting ventilation rate (McGhee *et al.*, 2013). A promising area for additional research is individual variation in foraging modes. In many species, individuals will switch between actively seeking prey or lying in wait to ambush prey that come near (McLaughlin, 1989). In fishes, the tendency for an individual to choose one mode over another is affected by environmental conditions (Fausch *et al.*, 1997; Killen *et al.*, 2007), and it is possible that the environmental thresholds causing a change in strategy may vary among individuals according to their metabolic traits. Individuals with high maintenance requirements, for example, may more readily switch to an active foraging mode for increased prey intake with modest increase in the costs of locomotion (Killen *et al.*, 2007).

### Habitat selection

Given recent interest in the potential effects of AS on the geographical distribution and range of species and populations (Pörtner & Farrell, 2008; Clark *et al.*, 2013), it is surprising that more work has not been done to determine whether metabolic traits influence the habitat preferences of individuals in response to spatial or temporal variation in factors such as temperature or hypoxia. As an example of this approach, the SMR of individual *P*. *phoxinus*, as measured at a single temperature, has been shown to exhibit a negative correlation with chosen temperature in a shuttle‐box apparatus (Killen, 2014). It is possible that individuals with higher maintenance requirements select lower temperatures in order to increase excess energy that can be allocated to growth, or to reduce the costs of activity and digestion to increase available AS, although the extent to which energy is freed up by moving to a lower temperature will depend on whether SMR drops faster with decreasing temperature than does MMR. Differences in conspecific density, predator density, food availability or structural complexity among habitats complicate this picture (Chabot & Guénette, 2013), so could cause individuals with different metabolic traits to prefer different habitats, either directly or *via* correlations with traits such as boldness. An example is juvenile *S*. *salar*, in which individuals with a lower SMR spend more time in covered areas (Finstad *et al.*, 2007a).

### Links to swimming performance

It is commonly assumed that individuals with a higher MMR or AS should have an increased capacity for aerobic swimming performance, and this trend is generally supported when comparing among populations or treatments within a given study. There have been surprisingly few studies, however, to examine links between either MMR or AS and measures of swim performance among individuals within a species. AS was found to be positively correlated with critical swimming speed (*U*_crit_) in cod *Gadus morhua* L. 1758 (Reidy *et al.*, 2000). Marras *et al.* (2013) observed that for *D*. *labrax* there was a positive correlation between MMR and the maximum attainable swimming speed during a constant acceleration test, but neither MMR nor AS were correlated with any other measure of swim performance measured in that study including maximum speed during an escape response and gait transition speed (*i*.*e*. the speed at which a fish changes from steady aerobic swimming to anaerobic burst‐and‐coast swimming). A reworking of the data presented in Killen *et al.* (2012a) reveals that for juvenile golden grey mullet *Liza aurata* (Risso 1810), AS is positively correlated with gait transition speed (Pearson correlation, *r* = 0·53, *P* < 0·05), but SMR is not related to either MMR, AS or gait transition speed.

Little is currently known of how intraspecific differences in aerobic (*e*.*g*. *U*_crit_ and gait transition speed) or anaerobic (*e*.*g*. fast‐start escape response and maximum sprint speed) swimming performance affect the behavioural ecology of individuals. Differences in swimming ability among individuals affect their spatial positioning within a school, individuals with a relatively high AS and gait transition speed may occupy anterior positions when the school is moving at a relatively high swim speed (Killen *et al.*, 2012a). An increased AS could allow individuals to swim at high speeds at the front of a school while simultaneously performing other physiological tasks, such as feeding or digestion. Intrinsic differences in swimming ability could be linked to intraspecific differences in migration success (Eliason *et al.*, 2011) or likelihood of escape from predation attempts (Walker *et al.*, 2005), but both of these topics require further study at the level of the individual. It is also noteworthy that individuals with a higher AS are also able to recover faster after bouts of exhaustive exercise (Marras *et al.*, 2010; Killen *et al.*, 2014). It is therefore possible that these individuals could resume normal foraging or anti‐predator behaviours sooner following intense exercise (*e*.*g*. after a predator attack), but this also requires further investigation.

## Priorities for future research

The increasing research focus on intraspecific variation has provided unprecedented insight into how individuals vary in the acquisition and utilization of energy, and how these differences affect relationships between physiology and behaviour. In many respects, however, this line of research is still in its infancy. Here, five gaps in the current literature are outlined that are considered to be of high priority for achieving a further understanding of the ecological and evolutionary importance of individual variation in metabolic traits in fishes: (1) Are MMR and AS linked to the behaviour of individual animals? Until recently, studies examining co‐variation in metabolic traits and behaviour in fishes have exclusively examined the role of SMR or some index of resting metabolic rate. MMR and AS could theoretically impose constraints on the simultaneous execution of aerobically fuelled behaviours and physiological tasks, and variation in MMR or AS among individuals could therefore also be linked to differences in activity, boldness or aggression. Knowledge of such relationships will be crucial for an integrative understanding of the adaptive value of different metabolic phenotypes (note that AS is generally more related to MMR, and hence sensitive to variation in MMR, than it is to SMR). The optimal combination of SMR, MMR and AS in different contexts (*e*.*g*. during food deprivation, hypoxia and temperature change) is unknown. Another important area for future study is the determination of how often routine behaviours actually cause fishes to approach their MMR, and whether this varies among individuals (Murchie *et al.*, 2011; Seebacher *et al.*, 2013; Killen *et al.*, 2014). Individuals that operate near MMR more frequently could be less able to cope with factors that decrease available AS, *i*.*e*. limiting and masking factors described by Fry (1971). (2) How does the environment modulate links between metabolic traits and behaviours? There is wide variation among studies with regard to the direction and magnitude of the associations between metabolic traits and behaviours. It is likely that the nature of these links are context dependent and labile in response to changes in environmental conditions, although only a handful of studies have investigated this issue in fishes (Killen *et al.*, 2013). This is a major area requiring further study, because the degree of selection on correlated traits could vary substantially in response to subtle changes in environmental conditions, especially if factors such as temperature, oxygenation or salinity alter the repeatability of metabolic traits. Further, depending on context, different metabolic traits may be linked to different aspects of behaviour. Food limitation, for example, may accentuate links between behaviour and SMR *via* differences in energy demand among individuals or effects of mass loss on behaviour. Situations that place demands on swimming performance or constrain the ability to perform physiological multi‐tasking by reducing AS (*e*.*g*. exposure to hypoxia) could generate correlations between MMR or AS and behaviour. (3) Does the metabolic phenotype influence habitat selection? Although it is often assumed that metabolic traits affect the habitat preferences of fishes in response to factors such as temperature and oxygen availability (Pörtner & Knust, 2007; Pörtner & Farrell, 2008), to date this possibility has not been thoroughly studied at the level of the individual in either the laboratory or the field. Recent technological advances in telemetry have allowed the tracking of individual fish in the natural environment at temporal and spatial scales not previously possible (Cooke *et al.*, 2013; Metcalfe *et al.*, 2016). This work has revealed intraspecific variation in habitat use and movement patterns (Kobler *et al.*, 2009), and an exciting next step in this line of research will be to determine if this variability is related to individual differences in traits such as SMR, MMR or AS. (4) How do SMR MMR, and AS correlate among individuals? Only a small number of studies have investigated correlations among SMR, MMR and AS among individuals, and these have shown species‐specific (or possibly context‐specific) variation in the direction and strength of these correlations. More work on this topic could reveal fundamental links among metabolic traits that constrain plasticity or evolutionary responses to environmental change. If an increase in MMR also requires an investment in SMR (to cover the maintenance costs of metabolic machinery), acquiring an elevated MMR may not be possible in all ecological niches or environments. Furthermore, there have been surprisingly few studies that directly examine links among either SMR, MMR or AS and swimming performance at the individual level. Given that swimming ability could affect fitness through a variety of mechanisms (*e*.*g*. foraging ability, predator avoidance, migration, mating success and parental care), then selection on swimming performance could result in correlated selection on metabolic traits in cases where such links do indeed exist. (5) What is the importance of metabolic traits for behavioural ecology beyond the juvenile stage? Studies of intraspecific diversity in metabolic traits in fishes have largely focussed on the early life stages, examining links between metabolic traits and growth rates or competition for territory after hatching or settlement. This is in contrast to work examining intraspecific variation in metabolic traits in other taxa (*e*.*g*. mammals and birds) where work is mainly performed with adults (Rosenfeld *et al.*, 2015). An advantage of studying early life stages is that selection has had little time to reduce the variation within the population, so making it easier to detect effects of metabolic rate on fitness, but there are at least three fundamental problems with this emphasis on juvenile organisms. Firstly, the reproductive period could be an important selective bottleneck and yet almost nothing is known about how variation in SMR, MMR or AS relate to reproductive success, mate choice and ability to provide parental care. Secondly, juveniles experience different costs and benefits associated with various behaviours as compared with adults (*e*.*g*. foraging in juveniles may come at a relatively high risk of predation) and so extrapolating links between metabolic rate and behavioural traits such as boldness or activity across life stages should be done with caution. Thirdly, juveniles are by definition growing organisms, and even though it is common practice to fast fish before measurement of SMR, prior food intake and growth trajectory can have lingering effects on behaviour, morphology and biochemistry that may confound estimates of SMR (Killen, 2014; Rosenfeld *et al.*, 2015).

## Conclusions

The extensive variation in SMR, MMR and AS among individual fish is to some degree repeatable, but the repeatability may diminish with time, perhaps because these traits are also sensitive to a range of environmental factors including feeding regime, temperature and oxygen availability. So far, only SMR has been thoroughly studied for links to foraging, predator avoidance and intraspecific aggression, and more work is needed to determine whether MMR and AS also show important associations with these categories of behaviour. The available evidence suggests that the environment may affect the degree of covariation between metabolic traits and behaviour. More knowledge in this area will be especially important for understanding responses to environmental change and what suites of traits comprising metabolic phenotypes (*i*.*e*. different combinations of SMR, MMR and AS) are most advantageous under a given set of conditions.

N.B.M. was funded by ERC Advanced Grant 322784, T.E.V.L. by EU Interreg IVA Programme project 2859 ‘IBIS’ and NSERC PGS‐D3 and S.S.K. by NERC Advanced Fellowship NE/J019100/1. This Special Issue paper was an invited contribution based upon decisions made within the EU COST Action FA1004, Conservation of Marine Fishes. We thank two anonymous reviewers for helpful comments that improved this paper.
